# Supplementary material for: Macrophage Cell Membrane Coating on Piperine-Loaded MIL-100(Fe) Nanoparticles for Breast Cancer Treatment
Source: J Funct Biomater. 2023 Jun 11;14(6):319. doi: 10.3390/jfb14060319 (PMC10299274; doi:10.3390/jfb14060319)
Supplement: Supplementary file 1 [file jfb-14-00319-s001.zip › jfb-2422138-supplementary.pdf]

SUPPORTING INFORMATION

Macrophage Cell Membrane Coating on Piperine-Loaded MIL-100(Fe) Nanoparticles for Breast Cancer Treatment

Christian Rafael Quijia[1], Geovana Navegante[2], Rafael Miguel Sábio[1], Valeria Valente[2], Alberto Ocaña[3], Carlos Alonso-Moreno[4], Regina Célia Galvão Frem[5], and Marlus Chorilli[1*]

[1] Department of Drugs and Medicines, School of Pharmaceutical Sciences of São Paulo State University (UNESP), Rodovia Araraquara Jau, Km 01 – s/n – Campos Ville, 14800-903 Araraquara, Sao Paulo, Brazil. E-mail: christianqui@hotmail.com; rafael.m.sabio@unesp.br; marlus.chorilli@unesp.br

[2]Laboratory of Molecular and Cell Biology, School of Pharmaceutical Sciences, Department of Clinical Analysis, São Paulo State University (Unesp), Araraquara, Brazil. E-mail: geonavegante@gmail.com; valenteval@gmail.com

[3]Experimental Therapeutics Unit, Hospital Clínico San Carlos, IdISSC, Fundación Jiménez Díaz, START, 28040 Madrid, Spain. E-mail: alberto.ocana@salud.madrid.org

[4]Unidad NanoDrug, Facultad de Farmacia, Universidad de Castilla-La Mancha, 02008 Albacete, Spain. E-mail: Carlos.AMoreno@uclm.es

[5]Institute of Chemistry, São Paulo State University (UNESP), Prof. Francisco Degni 55, 14800-060 Araraquara, Sao Paulo, Brazil. E-mail: rcgfrem@gmail.com

[*]Corresponding author at Department of Drugs and Medicines, School of Pharmaceutical Sciences of São Paulo State University (UNESP), Rodovia Araraquara Jau, Km 01 – s/n – Campos Ville, 14800-903 Araraquara, Sao Paulo, Brazil. E-mail address: marlus.chorilli@unesp.br

HPLC assay for PIP

The calibration curve was linear by analyzing a series of PIP concentrations in ethanol from 6.25 to100 μg mL^{-1} with a correlation coefficient of R^2=0.990 (**Figure S1**). The limit of detection (LOD) and limit of quantification (LOQ) were found to be 0.25 and 0.77 μg mL^{-1}, respectively. Standard samples were prepared and injected in triplicates on three successive days.

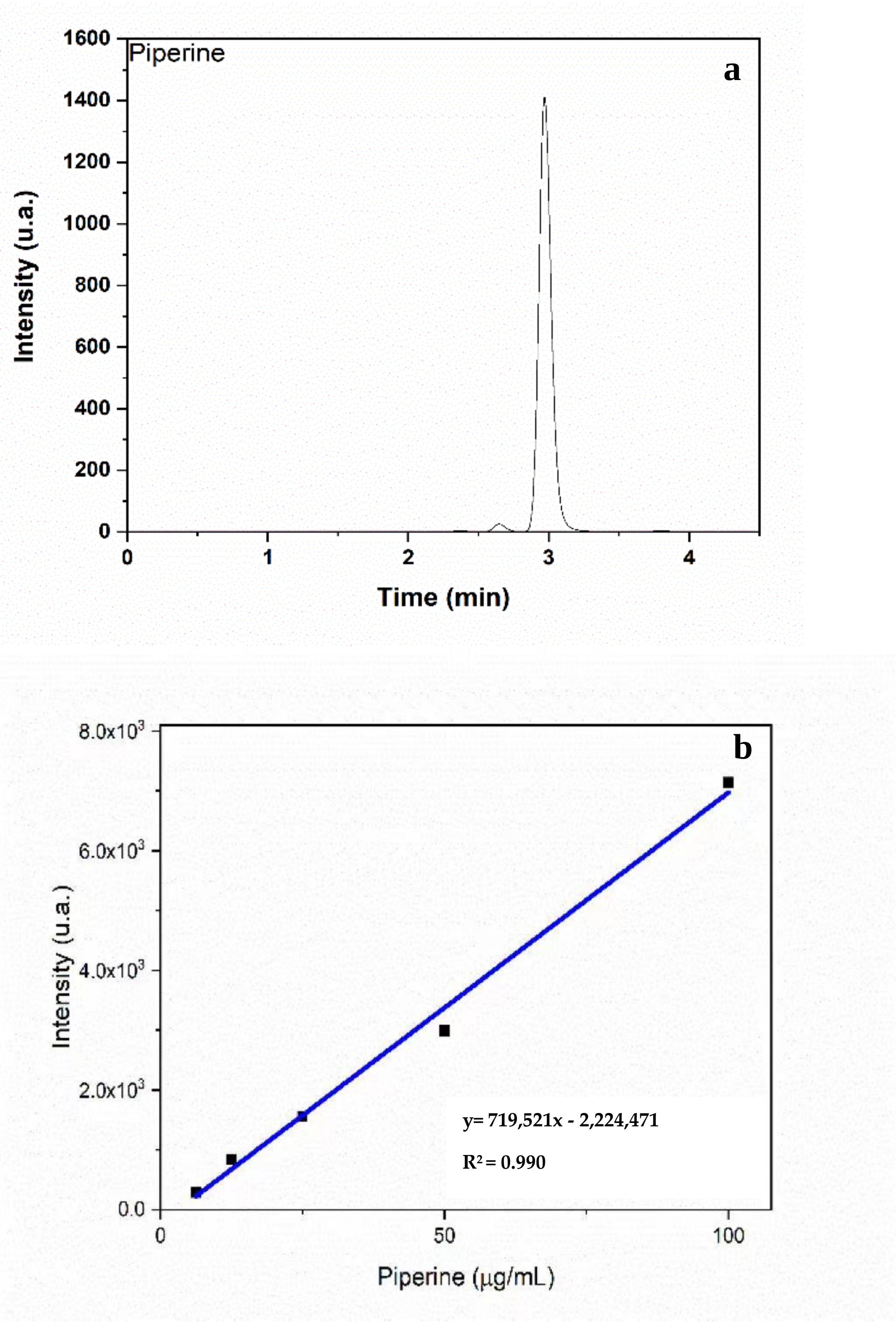

Figure S1. (**a**) Chromatogram of PIP (concentration = 6.25 to 100 µg mL^{-1}) in mobile phase (methanol/water (75:25)) and (**b**) calibration curve of PIP quantified by HPLC.

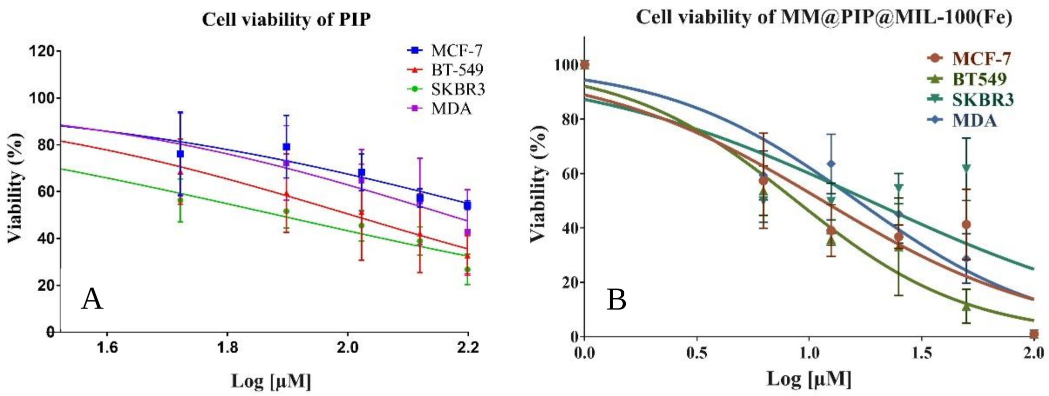

Figure S2. Cytotoxic activity of piperine (A) and MM@PIP@MIL-100 (Fe)- (B). The results represented cell viability and refer to the averages of three independent experiments (mean ± standard deviation). Cells were treated with 6.25 to 100 μM and incubated for 48 h. Viability was analyzed by nonlinear regression in GraphPad Prism version 7.0 (GraphPad Software, San Diego, CA, USA).

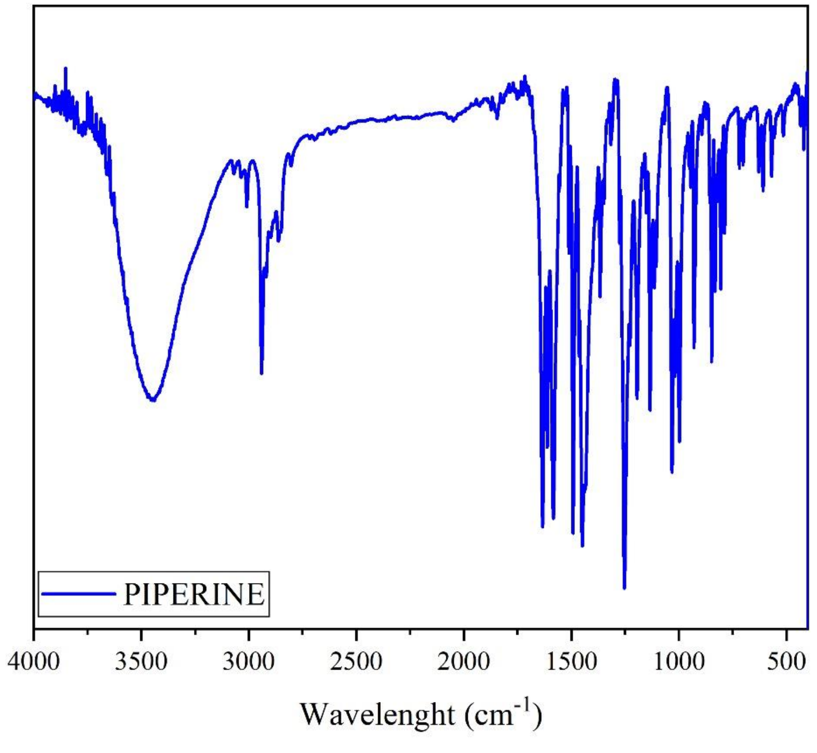

Figure S3. Fourier-transform infrared (FT-IR) spectrum analysis of PIP (blue line).

Synthesis of MIL-100(Fe)

The synthesis of MIL-100(Fe) was performed using a previously established method. [1] In this method, iron (III) chloride hexahydrate (0.081 g; 0.29 mmol) and BTC (0.028 g; 0.13 mmol) were dissolved in distilled water (1 mL). The resulting mixture was heated to 130 °C for 30 seconds and maintained at this temperature for 7 minutes (300 W) using a Mars-5 microwave reactor (CEM, US) with a maximum power output of 300 W and a frequency of 60 Hz. The MIL-100(Fe) product was obtained by centrifugation at 5600g for 8 minutes. To activate the solid, it was redispersed in absolute ethanol (1 mL) and subjected to centrifugation at 5600 g for 8 minutes. This process was repeated for a total of 4 cycles. The resulting orange solid was recovered with a yield of 98% (40 mg). Finally, the nanostructure was stored in pure ethanol at 4 °C for preservation.

PIP@MIL-100(Fe) in situ

We conducted the microwave synthesis of PIP@MIL-100(Fe) on-site following a previously described method, but with a variation. In this case, we introduced different concentrations of PIP (1, 4, 18, and 38 mg) into the initial synthesis solution. After the synthesis, the resulting PIP@MIL-100(Fe) was separated by centrifugation at 5600g for 8 minutes, and the sample with the most favorable chemical properties was chosen for further investigation. To continue the research, the recovered PIP@MIL-100(Fe) was then dispersed again in absolute ethanol (1 mL), subjected to centrifugation at 5600g for 8 minutes, and this process was repeated four times. As a result, an orange solid (40 mg, 96% yield) was obtained. To maintain the integrity of the nanostructure, it was stored in absolute ethanol at a temperature of 4 °C until it was ready to be used (see Fig. S4).

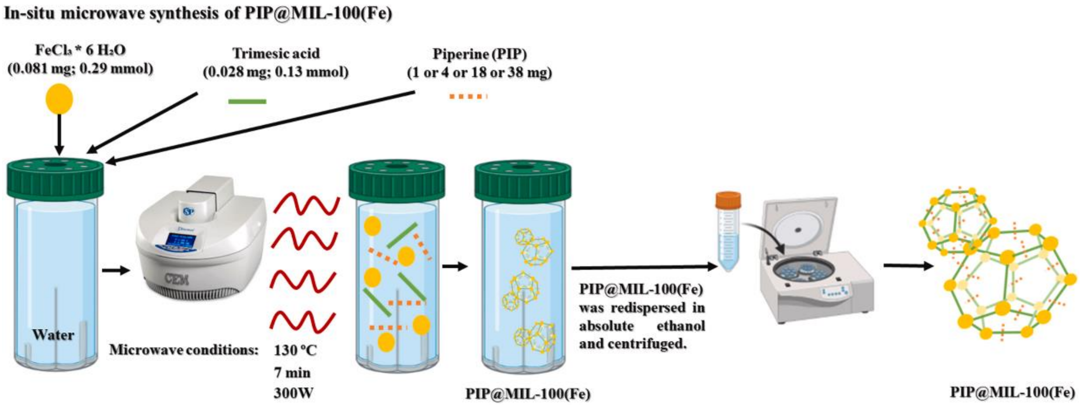

Figure S4. We performed the in-situ microwave synthesis of PIP@MIL-100(Fe) by dissolving BTC (0.028 mg; 0.13 mmol), iron (III) chloride hexahydrate (0.081 mg; 0.29 mmol), and various concentrations of PIP (1; 4; 18; and 38 mg) in 1 mL of distilled water. The reaction mixture was then subjected to microwave irradiation at 300 W, heating it to 130 °C for 30 seconds, and maintaining this temperature for 7 minutes. Next, the obtained PIP@MIL-100(Fe) was dispersed again in 1 mL of absolute ethanol, followed by centrifugation at 5600 g for 8 minutes. This centrifugation and redispersion process was repeated four times. As a result, an orange solid (40 mg, 96% yield) was recovered.

REFERENCE BIBLIOGRAPHIC

[1] C. R. Quijia *et al.*, "In situ synthesis of piperine-loaded MIL-100 (Fe) in microwave for breast cancer treatment," vol. 75, p. 103718, 2022.
